# Time to Move Again: Does the Bereitschaftspotential Covary with Demands on Internal Timing?

**DOI:** 10.3389/fnhum.2016.00642

**Published:** 2016-12-21

**Authors:** Rolf Verleger, Mechthild Haake, Alexandra Baur, Kamila Śmigasiewicz

**Affiliations:** ^1^Department of Neurology, University of LübeckLübeck, Germany; ^2^Institute of Psychology II, University of LübeckLübeck, Germany

**Keywords:** Bereitschaftspotential, readiness potential, movement, time

## Abstract

When *Bereitschaftspotentials* (BPs) are measured, participants are required to voluntarily perform a predefined number of identical movements, with varying intervals between movements, exceeding some obligatory minimum interval. Participants might cope with these demands on timing by installing a slow, broadly tuned rhythm of activation, serving as an internal trigger for executing movements in time. The BP might reflect the rising phase of this activation, culminating at the movement. If so (i) not only should BP amplitudes become larger, but BPs should also have their onsets earlier before movements when longer minimum intervals are required between movements (Experiment 1). Further, (ii) BP amplitudes should covary with demands on internal timing: decrease when internal timing is less necessary and increase in the other case. Variation of timing demands was realized by requiring participants to count vs. not to count the seconds between movements (Experiment 1) and by regular vs. irregular vs. no ticking of a clock (Experiment 2). Prediction (i) was confirmed while prediction (ii) was not. Thus, BP onsets did vary in accordance with the temporal constraints about when the movements should be performed, suggesting some relation to timing mechanisms, but we could not provide evidence for the notion that the process reflected by BPs is this timing mechanism.

## Introduction

The Bereitschaftspotential (BP; “readiness potential”, RP) is measured by requiring participants to perform a simple movement (e.g., bending their index finger) whenever they wish, recording EEG in the time epochs before each of these movements, and averaging EEG across these repetitions (Kornhuber and Deecke, 1965, 2016). The BP consists of two parts (Kornhuber and Deecke, 1965; Shibasaki and Hallett, 2006): The early phase (BP proper, “early BP”) is a slow, non-lateralized negative shift, probably generated in the supplementary motor area (SMA) and anterior neighboring areas (pre-SMA and anterior mid-cingulate cortex; Deecke and Kornhuber, 1978; Deecke et al., 1987; Ball et al., 1999; Cunnington et al., 2002, 2003; Shibasaki and Hallett, 2006; Nguyen et al., 2014). In the late phase (about 400 ms before the movement) this BP proper is overlapped by lateralized potentials that reflect activity of the motor cortex controlling the moving limb (Kornhuber and Deecke, 1965; Kutas and Donchin, 1980).

As is well known, discovery of the BP has prompted vivid debates about the relationship between brain and mind, focused on the question whether the brain’s movement preparation reflected by the BP precedes, or depends on, conscious intention (e.g., Libet, 1985; Deecke and Kornhuber, 2003; Navon, 2014; Deecke and Soekadar, 2016; Schultze-Kraft et al., 2016). In these debates, it remained beyond doubt that the BP reflects a process necessary for movement preparation.

This interpretation is straightforward since participants are required to perform a movement while nothing else happens. However, like all event-related EEG potentials, the BP is measured as average potential, obtained by multiple repetitions of the same event. Therefore, what participants are actually required to do in BP studies is to execute the same movements in multiple repetitions (e.g., *n* = 60, or even *n* = 512 in Kornhuber and Deecke, 1965). Specific to the BP situation is that these repetitions occur without external timing and under the explicit instruction to use irregular intervals between the movements. But the absence of explicit external timing does not mean absence of timing requirements. These requirements are that movements are expected to be performed no sooner after the preceding movement than, e.g., 3 s (Shibasaki et al., 1980; Wessel et al., 1994) or even 15 s (Kornhuber and Deecke, 1965; because the measurement interval before movements should be free of any past activation) and that the experiment should be over after some time, meaning that participants should not wait endlessly until the next movement. This leads to the hypothesis that, notwithstanding some outlying values, participants in typical BP studies will restrict the intervals between movements to some range, which might have been, e.g., 20–40 s in Kornhuber and Deecke (1965) study (i.e., well over the required minimum interval of 15 s but not endlessly) or “once every 5 s or longer” as Shibasaki and Hallett (2006) put it in their review (p. 2342). Unfortunately, the actual intervals between movements have hardly ever been measured and reported in studies using the classical BP paradigm without any external stimulation (in contrast to studies using Libet et al., 1983, task with the rotating clock hand). Nor have the effects of different timing demands on the BP been investigated in a graded, systematic fashion. For example, Kunieda et al. (2000) made subdural recordings from motor areas in four epilepsy patients and compared a BP situation, movements being required in a self-paced manner at about once every 5 s, to a non-BP situation, with rhythmic continuous movements at a rate of once every 500 ms. Activation of SMA and pre-SMA, probably equivalent to scalp-recorded BPs, was found in the BP situation only. Much variation is possible between intervals of 5 s and 500 ms and has remained largely unexplored so far.

When faced with this requirement of repeating the same movement over and over again in some loosely defined time-window, participants might install, and then rely on, some internally generated fuzzily defined rhythmical activity. Periodically, this activity might be initiated and then increase until some subjective threshold is reached which is taken by the participant as an optional prompt to perform the movement. The BP may represent this activity. Indeed, relations between both SMA and pre-SMA and time estimation have been well established in fMRI studies (Schubotz et al., 2000; Coull et al., 2004, 2015) and have also been suggested long ago with respect to the relation between (pre-)SMA and BP (Deecke et al., 1985). There is also impressive work on timing characteristics of SMA neurons in monkeys, most recently in a series of studies by Merchant and colleagues where monkeys had to continue a regular beat (e.g., Merchant et al., 2011, 2015) although in intervals ≤1 s, i.e., well below the intervals used in BP studies, and the intervals were explicitly regular, in stark contrast to the BP situation. More similar to the BP situation was a study by Lebedev et al. (2008) where monkeys had to press a button for 2.5–4.5 s and then release it. This variable interval before the act of releasing may be considered as defined in a similarly temporally fuzzy way as the act of moving in the BP situation and, indeed, the time-course of firing of neurons in pre-motor and motor cortex bore similarity to the time-course of the BP. Moreover, again in humans, the BP has been shown to increase with the presence of explicit demands on timing (Baker et al., 2012).

Most to the point, the contingent negative variation (CNV) has been shown to be closely related to timing requirements. The CNV (first described by Walter et al., 1964) is a slow negative potential developing before an expected stimulus, culminating at stimulus onset. Usually, the expected stimulus requires a response. Therefore, some authors have suggested that the late part of CNV is actually a BP (e.g., Rohrbaugh et al., 1976). On the other hand, the response in the CNV situation is externally triggered whereas the action in the BP situation is the product of some internal decision. In line with this, others have emphasized dissociations between these two components (e.g., Ikeda et al., 1994, 1997; Cui et al., 2000a; Deecke, 2014). It seems plausible to assume that BP and late CNV share some core activity (van Boxtel and Brunia, 1994; Cui et al., 2000a; Verleger et al., 2000), related to their common generators in the mesial (supplementary and cingulate) motor areas (Cui et al., 2000a,b). This core activity might be movement preparation, as usually assumed, but alternatively it might be related to timing-related factors, like time estimation (Casini and Vidal, 2011) or temporal expectancy (van Rijn et al., 2011), in line with the above-mentioned function of the SMA and with several studies that explicitly relate late CNV to temporal processing (Macar et al., 1999; Trillenberg et al., 2000; Mento et al., 2015; Faugeras and Naccache, 2016).

Interesting in this context is the variability of BP onsets between studies and between or within participants (though see the good reproducibility of the BP waveform in a single participant’s recordings from eight sessions in Deecke et al., 1976). Quite often, the BP rises immediately at the beginning of the analyzed and graphically presented period, be it 1.5 s (Libet et al., 1982, 1983) or 2.5 s (Haggard and Eimer, 1999; Jo et al., 2014) such that the question must be asked whether this earliest recorded time-point, long before the movement, is indeed the time where the BP begins. Why do participants need more than 1 s to decide on a simple movement? More generally, it may be asked whether a time-point when the BP begins can be defined at all or whether the BP is part of long-lasting shifts of activation (Schurger et al., 2012; Jo et al., 2013). Being well aware of this problem, Libet et al. (1982, 1983) distinguished between several BP types, with the frequent “Type 1” representing BPs without clearly defined onset. If the BP reflects movement preparation, it is not easy to understand why it sometimes does and sometimes does not start more than 2 s before a simple repeating unchanged movement.

Therefore, to test the effects of temporal parameters on the BP, the temporal constraints that are implicit in BP tasks were explicitly varied in the present study. To detail, in Experiment 1 participants were instructed to press the mouse key at freely chosen intervals, but no sooner than at some minimum interval. This minimum interval varied between blocks from 1 s to 5 s. If reflecting brain activity needed to perform spontaneous movements, BPs will not be affected by this variation of intervals and always start at a constant time before movements. In contrast, if reflecting a slow rhythm implemented by participants to deal with the temporal constraints of the BP task, the BP will increase in width, starting earlier and earlier with increasing minimum intervals. These different predictions about BP onsets may also be studied by analyzing the restarting BPs after preceding movements. If reflecting a slow rhythm to deal with the temporal constraints, BPs will start at a constant time after preceding movements, independently of the required minimum interval, and will have reached constant amplitudes after about 1 s (before the first new movements start in the shortest, 1 s minimum interval). In contrast, if reflecting brain activity needed to perform spontaneous movements, BPs’ restarts will depend on the required minimum interval, starting later when the next movements will take place later.

If indeed reflecting a means implemented by participants for coping with the timing requirements in a temporally unstructured situation, BPs might become unnecessary and, therefore, will decrease in amplitude when time is structured by other means. One of these means may be counting the seconds. The requirement not to count the seconds appears to be standard in BP research (explicitly mentioned, e.g., by Matsuhashi and Hallett, 2008; Misirlisoy and Haggard, 2014) probably out of concern that participants might not produce an autonomous, free-will intention to act when acting is triggered by their mental count. We are not aware of any study that tested BPs for the effects of this instruction. Therefore, participants were asked not to mentally count in one part of Experiment 1 and, to the opposite, instructed to mentally count the seconds of the required minimum time in the other part. We assumed that BP amplitudes would decrease with counting because the timing mechanism reflected by BPs would be less needed when temporal information is provided by counting. In Experiment 2, we provided an external timing aid by presenting a steady beat of two ticks per second. Again, we assumed that BP amplitudes would decrease compared to the standard condition because the timing mechanism reflected by BPs would be less needed. In contrast, in the *erratic* *clock* condition, the beat of tick-tocks changed after each movement. We assumed that the BP would increase in this condition, due to the requirement to enforce internal timing against external interference.

## Material and Methods, Experiment 1

### Participants

Twenty-five healthy volunteers were recruited. Written informed consent was given by all participants. Due to artifacts (*n* = 5) and failure to follow instructions (*n* = 4; see below), the data of nine individuals had to be removed. The 16 remaining participants (8 men, 8 women) had a mean age of 25 years (SD = 3 years) and were right handed (mean of Edinburgh Handedness Inventory score = 76%). The study was approved by the University of Lübeck ethics committee.

### Stimuli and Procedure

Participants sat comfortably in front of a 1.2 m distant computer screen. The session consisted of eight blocks, initiated by the experimenter. During these blocks, participants watched a black fixation cross at the center of the light-gray 16″ screen. They were asked to sit quietly and press the left mouse key with their right index finger 50 times during each block, plus three times at the start of each block for practice. The time-point of pressing could be freely chosen, with only one constraint, announced before each block on the screen and by oral instruction: the minimum time interval that had to pass between two key-presses. This minimum interval varied across blocks between 1 s, 2 s, 3 s or 5 s. There was no upper limit set on these intervals. Participants were made aware that an error message would appear if they pressed earlier than the minimum time interval, saying “pressed too early” in large red letters (in German, Helvetica 30 pt) for 4 s. After error messages, they had to wait anew for the minimum time before key-pressing. The fixation cross flickered briefly with each valid key-press, as feedback for a valid movement, by turning blue for 100 ms at 100 ms after pressing. The program Presentation (Version 17.0) was used to record the mouse clicks, present the fixation cross and the “too early” message, and send event codes to another computer that recorded EEG.

The session consisted of two parts. In one part, the intervals between key-presses had to be filled by mentally counting the seconds from the last press, and in the other part participants were instructed not to count. Either part consisted of four blocks, differing by the required minimum intervals which were presented either in ascending order (1 s, 2 s, 3 s, 5 s) or in descending order (5 s, 3 s, 2 s, 1 s). Half the participants had the counting blocks first, and half the no-count blocks, and within either group, half the participants had the minimum intervals in ascending order and half in descending order.

A questionnaire was given to all participants after they had finished the experiment. The questions concerned any problems with the task and if time estimation was more demanding whilst counting seconds mentally or without counting. Participants gave an estimate about the percentage of trials they were able to follow either instruction and named strategies used to avoid counting.

### EEG Recording and Preprocessing

EEG was recorded with Ag/AgCl ring electrodes fixed in a cap (Easycap, Herrsching/Germany^1^) from 60 scalp sites, including eight midline positions from AFz to Oz and 26 pairs of symmetric left and right sites. Additional electrodes were placed at the tip of the nose for offline reference and, as connection to the ground, at Fpz. Online reference was Fz. For artifact control, electrooculogram (EOG) was recorded, vertically (vEOG) from above vs. below the right eye and horizontally (hEOG) from positions next to the left and right outer rims of the eyes. 80% ethanol, a combined electrolyte and abrasive paste (Körner pharmacy, Graz, Austria) and cotton buds were used to clean hair and skin beneath the electrodes and to lower impedance <5 kΩ (reference and ground electrode <1 kΩ). To approach a steady electrochemical state, electrodes were allowed to settle for 10 min before recording. Using BrainVision Recorder software (version 1.20^2^) EEG signals were amplified from DC to 1000 Hz by BrainAmp MR plus amplifiers (Brain Products, Gilching, Germany), digitized with 500 Hz, and stored on hard disk. Offline processing was done with BrainVision Analyzer software (version 2.1^2^). EEG data were re-referenced to the tip of the nose (as in Kornhuber and Deecke, 1965; Haggard and Eimer, 1999), low-pass filtered at 20 Hz and divided into movement-locked epochs. As motivated in the “Introduction” Section, epochs were formed in two ways, assessing (in the usual way) how BPs developped until the movement and, furthermore, how BPs restarted after movements. For the time window focusing on pre-movement development, EEG was selected from 5 s before each key press (7 s for the 5 s minimum condition) to 1 s afterwards. For the time window focusing on post-movement restart, EEG was selected from the key press until 2.5 s afterwards. In either case, editing trials for artifacts included rejecting trials with gross artifacts when minimum and maximum of voltages in any EEG channel differed by more than 250 μV or when consecutive data points differed by more than 50 μV (except the 4 EOG electrodes and AF3, AFz, AF4, so that trials would not be rejected for blinks). The mean amplitude of the first 100 ms were used as preliminary baseline for the pre-movement potentials. For post-movement potentials, 700–900 ms after key-press were used as baseline (when the movement-evoked potential had subsided). Ocular artifacts (eye movement and blinks) were removed using the linear regression method implemented in Brain Analyzer. The baseline was adjusted after this step and segments rejected when voltages exceeded ±150 μV in any EEG channel. Trials were averaged separately for each condition. To remove artificial slow drifts in the pre-movement potentials, the first 100 ms and last 100 ms of the averages were compared for linear trend and the linear drift was subtracted from the data of each segment separately. Pooling across count and no-count instructions, the mean number of included trials in the pre-movement potentials was 80 per average, with a minimum number of 20 (Means ± SDs for 1 s, 2 s, 3 s, 5 s conditions: *n* = 85 ± 14, 84 ± 11, 82 ± 21, 69 ± 22, minima *n* = 51, 62, 43, 20). For the separate analysis of count and no-count conditions, two participants had too few trials in one of the conditions. Mean number of included trials in the remaining 14 participants was 41 per average, with a minimum number of 9 (Means ± SDs for 1 s, 2 s, 3 s, 5 s conditions in count instruction: *n* = 41 ± 9, 42 ± 6, 43 ± 8, 38 ± 10, minima *n* = 24, 32, 20, 11; in no-count instruction: *n* = 43 ± 10, 42 ± 10, 43 ± 11, 35 ± 13, minima *n* = 16, 24, 12, 9). Much more data survived artifact editing in the shorter post-movement potentials. Mean number of included trials was 49 per average, with a minimum number of 16 (Means ± SDs for 1 s, 2 s, 3 s, 5 s conditions in count instruction: *n* = 50 ± 3, 49 ± 4, 49 ± 4, 50 ± 4, minima *n* = 40, 38, 35, 39; in no-count instruction: *n* = 49 ± 6, 49 ± 6, 47 ± 8, 47 ± 9, minima *n* = 31, 27, 28, 16). Grand averages were formed over participants and low-pass filtered at 5 Hz for concise illustration of results.

### Data Analysis

To analyze button-press timing, medians, means, and standard deviations (SDs) of intervals between movements were determined for each participant in each condition. Four of the originally 25 participants had grossly outlying values in one or more conditions (median interval slower by more than 2 SDs of the other participants, or number of too early key-presses exceeding 2 SDs of the other participants) and were excluded from the study. Means and SDs of the remaining 16 participants (five others were excluded because of too few trials remaining after editing for EEG artifacts, as noted) were evaluated with analyses of variance (ANOVA, IBM SPSS, Version 22). Within-subject repeated measurement factors were Minimum Time (1 s, 2 s, 3 s, 5 s) and Count Instruction (count/not count).

To define BP parameters, averages were smoothed by low-pass filtering at 2 Hz (48 dB slope). BP amplitudes were measured as mean amplitudes 400–200 ms before the key-press against baselines, as defined below (BPs tended to reach their maximum at 300 ms before the key-press rather than at movement onset, most probably because, unlike in Kornhuber and Deecke, 1965, and many following BP studies, but like, e.g., in Haggard and Eimer, 1999, or Baker et al., 2012, time-points of movement onsets were here given by the key-press rather than by the onset of preceding muscle activation). BP onsets before movements were determined in the Cz waveforms as the latest time-point (but earlier than 400 ms before key-pressing) when amplitudes finally rose above 20% of the BP amplitude. These measures of BP amplitudes and onsets critically depended on the choice of baseline. Since the BP waveforms differed in the time-courses of their ups and downs between conditions by design, due to different intervals between movements, baseline epochs could not be fixed across conditions. What could be done is defining a fixed criterion across conditions: Baseline epochs were most plausibly defined as the lowest point reached by the waveforms between two movements. Therefore, these lowest (most positive) points were searched in a window that started after the average interval between movements. These average intervals were 1.8 s, 2.8 s, 4.3 s, 6.8 s for the 1 s, 2 s, 3 s, 5 s minimum conditions. Therefore, baselines were defined as the most positive value in a window 1.8–0.4 s before keypress onset in the 1 s minimum condition, 2.8–0.4 s in the 2 s, 4.3–0.4 s in the 3 s, and 6.8–0.4 s in the 5 s minimum conditions. BP restarts after movements were measured in the post-movement averages. Baseline was fixed at mean amplitudes 0.7–0.9 s after movements, and the restart was measured as mean amplitudes of 100 ms at 1.4 s and at 2.4 s after movements. These parameters were analyzed with ANOVAs with the repeated measurement factors Recording Site (the midline channels FCz, Cz, CPz, Pz where BPs were largest), Minimum Time (1 s, 2 s, 3 s, 5 s), Count Instruction (count/not count) and, in post-movement potentials, Epoch (1.4 s, 2.4 s after movement). In the pre-movement potentials, because of too few trials in single cells of this design for two participants, data were pooled across count instructions for analysis of the Minimum Time effect. These two participants were then omitted when assessing the effect of Count Instruction in the full three-factorial design.

To interpret interactions, ANOVAs were conducted separately for the levels of each of the interacting factors. Degrees of freedom of the Task and Recording Site factors (being repeated measures factors with more than two levels) were corrected with the Greenhouse-Geisser method.

## Materials and Methods, Experiment 2

Methods will be described only insofar as different from Experiment 1.

### Participants

Twenty healthy volunteers were recruited. Due to too many artifacts in their EEGs, data of four individuals had to be removed. The 16 remaining participants (7 men, 9 women) had a mean age of 26 years (SD = 2.1) and were right-handed (mean of Edinburgh Handedness Inventory score = 93%, range 60%–100%).

### Stimuli and Procedure

The session consisted of three blocks. Participants were instructed to press the left mouse key with their right index finger 100 times during each block at about 4 s after the preceding press. If this interval fell below 2 s, the “pressed too early” message was presented. The three conditions *no clock*, *steady* *clock*, *erratic* *clock* were presented separately, each in one block. Participants were informed about all three conditions at the onset of the experiment by written instructions on the screen, and the order of conditions was balanced across participants. The *no clock* condition was the standard situation of no external timing aid. In the *steady* *clock* condition, a steady rhythm of two beats per second was presented, like a clock’s ticking. Beats were produced by the computer’s sound card, using the Presentation software’s Audio-Space function. They had 50 ms duration and were presented by two loudspeakers (Hama AC-150), left and right of the screen with beat intensity set to 30 dB (measured by Sound Meter by Splend Apps). In the *erratic* *clock* condition, the beat of ticking changed with each key-press, varying randomly between 2, 3, 5, 6 ticks per 2 s (i.e., 1, 1.5, 2.5, 3 per second, averaging to 2/s) thereby providing an unreliable signal.

### EEG Recording and Preprocessing

Segments of EEG, recorded in the same way as in Experiment 1, were selected from 5 s before key presses to 1 s afterwards and were edited for artifacts in the same way as in Experiment 1. The mean number of included trials was 90 per condition, with a minimum number of 67 (Means ± SDs for *no*, *steady*, and *erratic clock* 5 s conditions: *n* = 89 ± 9, 91 ± 9, 90 ± 9, minima *n* = 70, 67, 74).

### Data Analysis

Button-press timing was analyzed in the same way as in Experiment 1. The within-subject repeated measurement factor for ANOVA was Clock (no, steady, erratic).

Since mean intervals between key-presses did not differ between conditions, the first 100 ms of the epochs (5.0–4.9 s before movements) were chosen as baselines of the pre-movement potentials in each condition. ANOVA factors for evaluation of BP amplitudes were Recording Site (FCz, Cz, CPz, Pz) and Clock (no, steady, erratic).

## Results, Experiment 1

### Movement Timing

**Figure 1:**
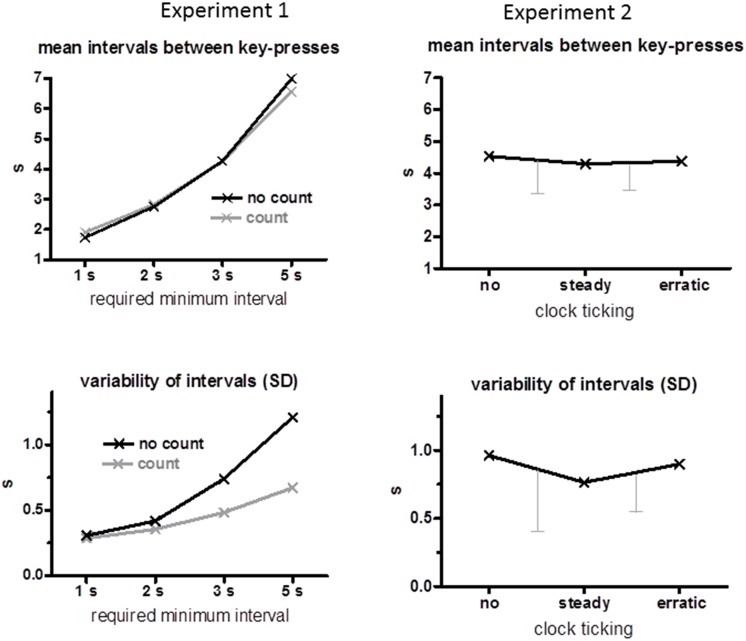
**Behavioral data.** Mean time intervals between key-presses are displayed in the upper panels and mean standard deviations across trials of these intervals are displayed in the lower panels. Data of Experiment 1 are compiled on the left, and of Experiment 2 on the right. The error bars of the Experiment 2 data are standard deviations of the differences between conditions, *no* minus *steady* and *steady* minus *erratic* (which is the relevant variability when conditions are compared within the same subjects, Cumming and Finch, 2005).

Mean time intervals between two key-presses and their mean intraindividual standard deviations are displayed in the left panels of Figure 1. As intended, mean intervals increased with increasing minimum time (*F*_(3,45)_ = 476.7, *p* < 0.001). There was no effect of the count instruction (*F* ≤ 2.3, *p* ≥ 0.13 for main effect and interaction). Likewise, variabilities increased with increasing minimum times (*F*_(3,45)_ = 32.5, *p* < 0.001), but much less so when intervals had to be counted (Count Instruction × Minimum Time *F*_(3,45)_ = 5.9, *p* = 0.02) such that variabilities were smaller for 3 s and 5 s minimum intervals when seconds were counted than when they were not (Count Instruction *F*_(1,15)_ = 11.4, *p* = 0.004; separately for 3 s *p* = 0.02, for 5 s *p* = 0.002).

### Questionnaire

Thirteen participants (81%) evaluated the no-count condition to be more demanding, two (12.5%) the count condition (*χ*^2^ = 8.1, *p* = 0.005), and one remained undecided. When instructed not to count, seven participants (44%) found orientation about timing in their breathing, four (25%) in some melody, three (19%) in some other sort of rhythm, two (12.5%) in some lyrics or sentences, five (31%) in something else, and two (12.5%) in nothing (More than one response could be given).

### Bereitschaftspotential

#### Effects of Minimum Interval

**Figure 2:**
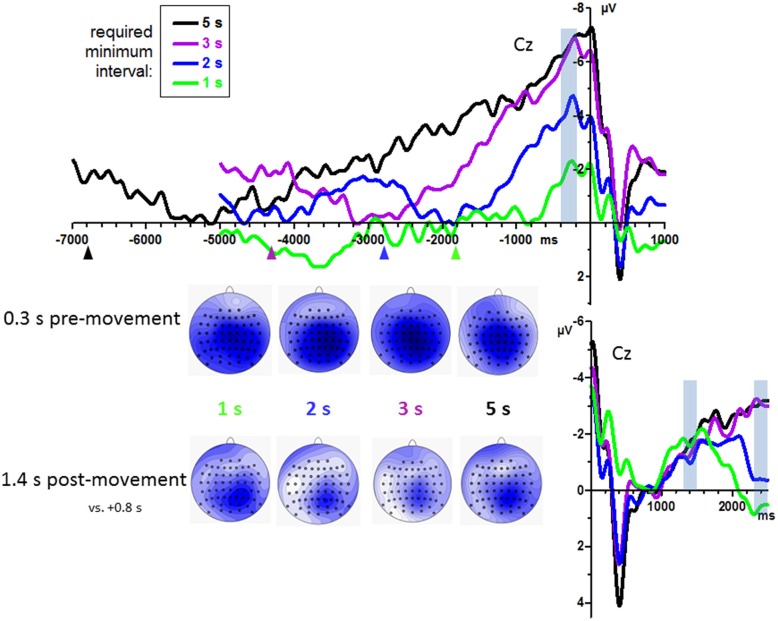
**Bereitschaftspotential (BPs) from Experiment 1: effects of required minimum interval.** Grand average waveforms (*n* = 16 participants) recorded at Cz. Time zero is the time of keypress both in the upper and in the lower panel. Upper panel: pre-movement time course. Lower panel: time course 2.5 s after movement onsets. Data have been pooled from count and no-count blocks. Transparent blue bars denote the time intervals of measurements. Colored triangles in the upper panel denote the average time interval of the preceding key-press, used here to form the left margin of the intervals in which the lowest (most positive) value was determined in each waveform as its baseline. Maps depict mean amplitudes during the indicated epochs. View of the head is from above, with Cz in the center and ear level (=120°) at the outer rim, nose is on top. Colors are min-max scaled in each map. Blue is negative, white is zero, positive would be red.

Grand mean waveforms are displayed in Figure 2, pooled across count and no-count conditions. BPs were analyzed at midline channels FCz, Cz, CPz, Pz, where they were largest as shown by the scalp maps in Figure 2. Among these sites, BPs tended to be smallest at FCz, *F*_(3,45)_ = 3.0, *p* = 0.06. Amplitudes differed between required minimum times, *F*_(3,45)_ = 26.2, *p* < 0.001 equally across recording sites, interaction *F*_(9,135)_ = 0.3, n.s., reflecting a linear increase across required minimum times, as indicated by the linear contrast *F*_(1,15)_ = 50.7, *p* < 0.001.

**Figure 3:**
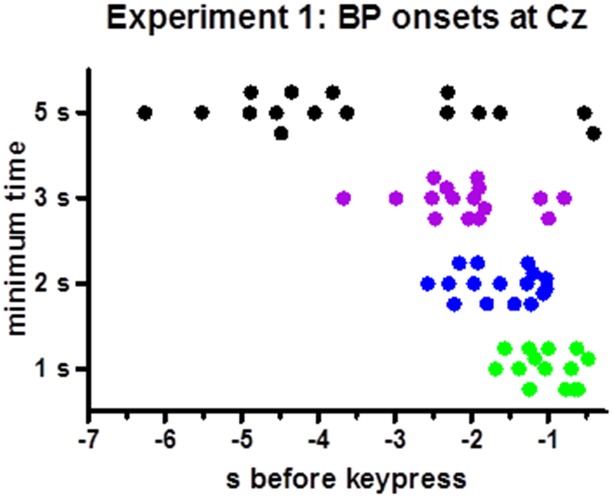
**Time points of individual BP onsets.** The figure shows the time points where 20% of peak-to-baseline amplitudes were finally exceeded, for each participant (Except for one participant in the 1 s minimum condition, where the BP peak was less negative than baseline such that no onset could be determined).

BP peaks were not more negative than baseline in one participants’ 1 s minimum waveform. Therefore, analysis of onsets was performed with 15 participants only. Individual onset times are displayed in Figure 3. Mean onsets at Cz were −1.0 s, −1.6 s, −2.2 s, −3.6 s relative to movement onsets for the 1 s, 2 s, 3 s, 5 s minimum times, *F*_(3,42)_ = 19.5, *p* < 0.001, with a significant linear contrast, *F*_(1,14)_ = 28.1, *p* < 0.001, reflecting reliably earlier onsets with 5 s minimum and later onsets with 1 s minimum than with each of the other minimum intervals (*t* ≥ 3.4, *p* ≤ 0.005 in pairwise comparisons) and a tendency for later onsets with 2 s than with 3 s minimum interval (*t* = 2.0, *p* = 0.06).

BP development after movements was measured at 1.4 s and 2.4 s by an ANOVA with the factors Epoch, Minimum Time, Counting, and Recording Site. BPs were larger at posterior than anterior sites (CPz > FCz; Recording Site: *F*_(3,45)_ = 5.0, *p* = 0.01). Importantly, BPs did not differ between minimum-time conditions at 1.4 s but did so at 2.4 s, indicating common onsets of the BPs and later divergence. This result was reflected by the interaction of Epoch × Minimum Time, *F*_(3,45)_ = 6.8, *p* = 0.001, and ensuing separate analyses of the effects of Minimum Time at 1.4 s (*F*_(3,45)_ = 0.3, n.s.) and at 2.4 s (*F*_(3,45)_ = 3.3, *p* = 0.048).

#### Effects of Counting the Seconds

**Figure 4:**
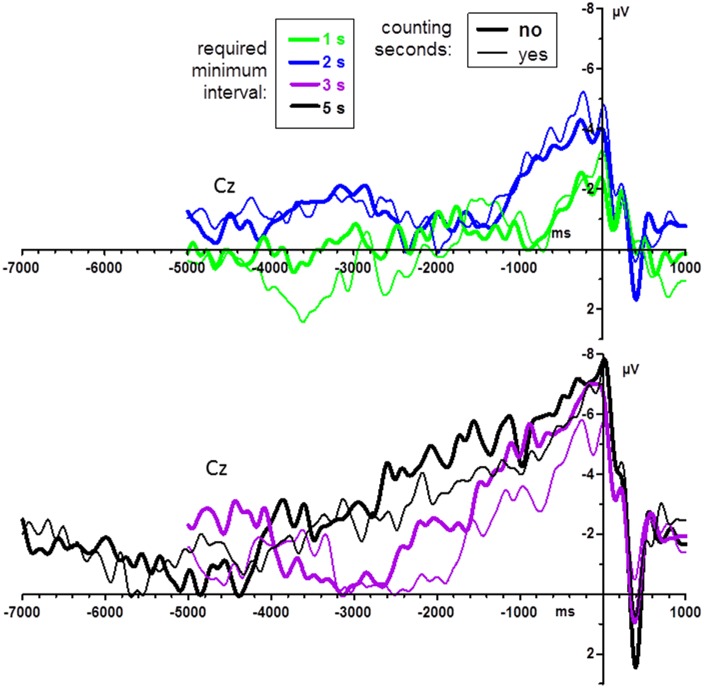
**BPs from Experiment 1: effects of instructions to count the seconds.** Grand average waveforms (*n* = 14 participants) recorded at Cz. Time zero is the time of keypress. For better visibility, data are shown separately for the shorter and longer minimum-time conditions (upper and lower panel, respectively). Bold lines denote the no-count condition, thin lines the count condition.

Counting vs. not counting did not have any significant effects in the last-mentioned analysis on post-movement potentials, all *F* ≤ 1.6, *p* ≥ 0.22. Pre-movement grand mean waveforms (*n* = 14) are displayed in Figure 4, separately for count and no-count conditions. As may be suggested from the figure, counting did not have any significant effects, neither on BP peak amplitudes, all *F* ≤ 1.7, *p* ≥ 0.22, nor on BP onsets, all *F* ≤ 2.1, *p* ≥ 0.16.

## Results, Experiment 2

### Movement Timing

Mean time intervals between two key-presses are displayed in the upper right panel of Figure 1. These intervals remained stable across clock conditions (no, steady, erratic, *F*_(2,30)_ = 0.5, n.s.) at 4.4 s on average. Variabilities of these intervals (lower right panel of Figure 1) decreased on average when the clock ticked steadily, but with large variability among participants, such that the difference between conditions did not attain significance, *F*_(2,30)_ = 2.1, *p* = 0.16.

### Bereitschaftspotential

**Figure 5:**
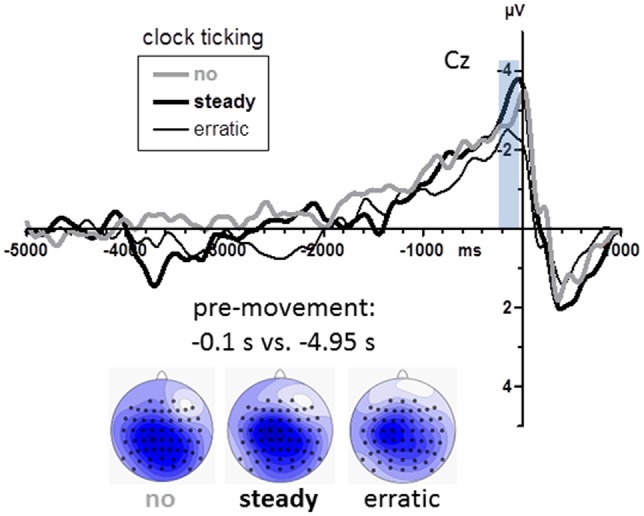
**BPs from Experiment 2.** Grand average waveforms recorded at Cz (*n* = 16). Time zero is the time of keypress. The transparent blue bar denotes the time intervals of measurements. Maps depict mean amplitudes during the BP peak intervals (0.2–0.0 s before movements) vs. baseline (4.9–5.0 s before movements). View of the head is from above, with Cz in the center and ear level (=120°) at the outer rim, nose is on top. Colors are min-max scaled in each map. Blue is negative, white is zero, positive would be red.

Grand mean waveforms are displayed in Figure 5. BPs were again analyzed at midline channels FCz, Cz, CPz, Pz (see scalp maps in Figure 5). Among these sites, BPs were equally large (Recording Site *F*_(3,45)_ = 1.2, n.s.). Obviously (Figure 5), BPs were neither smaller with the steady clock nor larger with the erratic clock than in the no-clock control condition. Accordingly, there were no effects of clock conditions (main effect and interaction with Recording Site *F* ≤ 1.6, *p* ≥ 0.20).

#### Subgroups

**Figure 6:**
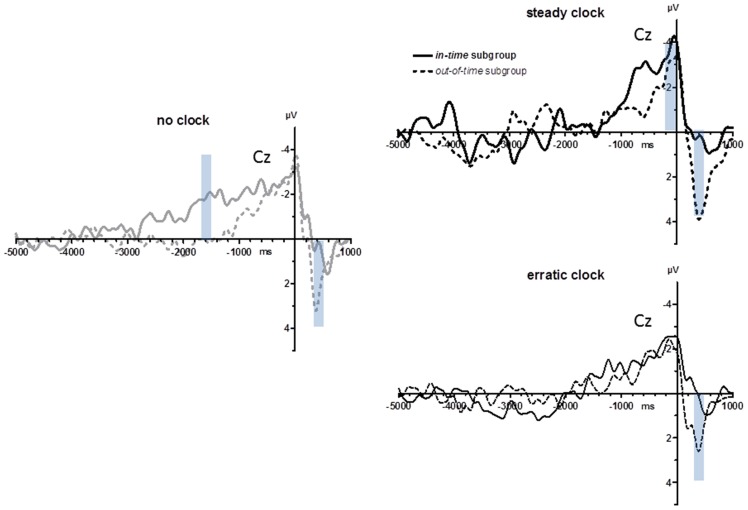
**BPs from Experiment 2: *in-time* vs. *out-of-time* subgroups.** Grand average waveforms recorded at Cz after a median split (*n* = 8 each) of the entire sample in subgroups who did (*in-time* subgroup) or did not (*out-of-time* subgroup) have decreased variabilities of intervals between movements in the *steady clock* relative to the *no clock* condition. Transparent blue bars denote the time intervals of measurements.

As displayed in Figure 1 (lower right panel) and noted above, variability of key-press intervals on average differed between the *no clock* and *steady clock* conditions, but by varying strongly across participants, this difference was not significant. In search of effects *post hoc*, we made a median split of this parameter, obtaining an *in-time* and an *out-of-time* group (*n* = 8 each): the *in-time* group behaved as expected, decreasing their variabilities from the *no clock* to the *steady clock* condition, whereas the *out-of-time* group did not. As a matter of course, these subgroups differed significantly in their defining parameter (Clock Condition × Subgroup in ANOVA on standard deviations of key-press intervals: *F*_(2,28)_ = 9.5, *p* = 0.002). Inspection of the subgroups’ grand means (Figure 6) suggests three possible differences: BP peaks might be largest with steady ticks in the *in-time* subgroup, BPs might rise earlier with no ticks in the *in-time* subgroup (around −1500 ms), and positivity evoked during movements might be smaller in the *in-time* than the *out-of-time* subgroup (at +400 ms). These *post hoc* observations were submitted to statistical testing on the two conditions *no clock* and *steady clock*. With regard to BP peaks, there was indeed an interaction of Clock × Recording Site × Subgroup (*F*_(3,42)_ = 3.5, *p* = 0.04) in agreement with the notion that the *in-time* group’s BPs were relatively largest with the *steady clock* at anterior sites FCz and Cz, yet this three-way-interaction could not be resolved to any significant two-way interactions and, therefore, could not be unambiguously interpreted. With regard to the effect at −1500 ms (mean amplitudes −1600 ms to −1400 ms) the interaction of Clock × Subgroup in ANOVA failed to reach significance (*F*_(1,14)_ = 4.3, *p* = 0.06; Nor was a significant interaction obtained when onset times were analyzed). In contrast, the post-movement positivity at 400 ms (mean amplitudes +300 ms to +500 ms) was indeed reliably larger in the *out-of-time* than the *in-time* subgroup (main effect of subgroup *F*_(1,14)_ = 9.1, *p* = 0.009) with additional topographical differentiation (Clock × Recording Site × Subgroup *F*_(3,42)_ = 4.1, *p* = 0.04) because these components were particularly large in the *out-of-time group* at anterior sites FCz and Cz in the *steady clock* condition (Clock × Recording Site in the *out-of-time* group *F*_(3,21)_ = 15.2, *p* = 0.001; in the *in-time* group *F*_(3,21)_ = 0.1, n.s.).

## Discussion

The aim of this study was to test to what extent the BP depends on timing. Our experiments were based on the idea that the BP reflects participants’ strategy of coping with the paradoxical message conveyed by the instruction that they were obliged to have a movement intention every few seconds. Specifically, we assumed that, in response to this instruction, participants generate a slow fuzzy fluctuation of activation reflected by the BP, to have an internal temporal guideline about when to move. Three consequent assumptions were tested: (1) with increasing prescribed minimum intervals between successive key-presses, the BP will increase in width, starting earlier and earlier relative to the key-press; (2) use of substitutes for the timing mechanism will tend to make the BP dispensable: Having participants explicitly count the seconds between key-presses or providing time information by an external clock will decrease the BP.

Hypothesis (1) was at least partially confirmed. When tested in the classical way introduced by Kornhuber and Deecke (1965) by looking backwards from movements, BPs had earlier onsets when the minimum interval was larger that was required to elapse between movements. If reflecting a brain process necessary and sufficient for movement, unrelated to timing, BPs should have started at the same time before movements. When tested in a new way, by looking forward from the movement, BPs developed in the same way after the foregoing movement, reaching a common negative level at 1.4 s. This level formed the BP peak for the 1 s minimum-interval condition and then decreased, remained on a plateau for the 2 s minimum-interval condition and later decreased, and further increased for the 3 s and 5 s minimum-interval conditions. If the BP would reflect a brain process necessary and sufficient for movement, unrelated to timing, it should start at staggered times after movements depending on the required minimum time intervals.

Also in line with hypothesis (1), BP amplitudes became larger when required minimum intervals were longer, with a highly significant linear contrast. This result may well be related to timing functions of the SMA (Schubotz et al., 2000; Coull et al., 2004, 2015; Merchant et al., 2011, 2015), in particular to the notion that the SMA has time-accumulator functions (Merchant et al., 2011). However, this result does not invalidate other accounts of the BP. In favor of the classical “movement preparation” notion, it may be argued that the “urge to act” (Libet, 1985) simply gets stronger with increasing time, or that our minimum intervals of ≤3 s are too small, such that the true BP simply needs more time to unfold which time is provided only with the 5 s minimum interval. Note that these arguments do not apply to the effect on onset latencies: The “urge to move” is expected to start at the same time after a preceding movement, irrespective of required minimum intervals.

It might further be argued (and actually was, by one reviewer of this article) that even the 5 s minimum interval is too short because true BPs may only be measured with minimum intervals of 15 s, as introduced by Kornhuber and Deecke (1965). Accordingly, our negative potentials would not be BPs but BP-like phenomena. We note that this criticism would apply to a large part of BP evidence reported in the literature, cf. Shibasaki and Hallett (2006) definition of “once every 5 s or longer”. Assuming a continuity between the present 5 s minimum (with actual average intervals of about 7 s) and a 15 s minimum condition, we would expect for an experiment comparing these two conditions that BP onsets will be earlier in the 15 s than in the 5 s condition.

In contrast, hypothesis (2) was not confirmed. We could not find any difference in the BP between the instruction to count the seconds and the instruction not to count. One reason for this might be that the numerous strategies reported by participants of Experiment 1 when they were required not to count (e.g., attending to breathing, imagined melodies, rhythms), might have qualities similar to mental counting, enabling participants to keep track of time.

However, it did likewise not make any clear difference whether an external steady beat helped participants in establishing the required interval of approximately 4 s between key-presses or whether such clock-ticking was absent. Moreover, when we made a *post hoc* distinction between participants who showed a positive effect of the external clock, by reduced variability between key-presses, and participants who did not show such an effect, the difference between these subgroups pointed to the direction opposite to what was predicted: In the participants with positive clock effects, i.e., who behaved according to our expectation, BPs tended to be larger in the *steady clock* than the *no clock* condition, rather than smaller. These results are not in line with the notion that the BP reflects a process related to timing.

Thus, the preliminary conclusion suggested by our two reported experiments is that BPs vary in accordance with the temporal constraints on the intervals between movements, but that the process reflected by BPs is not the timing mechanism itself.

As an aside, a clear difference between subgroups emerged in the positive potential evoked during movement onset which was generally smaller (independent of the clock condition) in the *in-time* subgroup whose variability was reduced by the external clock. This result suggests that these participants attended less than the *out-of-time* subgroup to something perceivable in the context of pressing, which might be the somatosensory reafference and/or the short flicker of the fixation cross. Thus, this result might be an event-related potential indicator of what was indicated by the *in-time* subgroup’s behavior, that they generally paid more attention to the clock ticking (which was not time-locked to movement onset and therefore did not evoke a distinct potential) than the *out-of-time* subgroup.

A number of methodological aspects deserve discussion, negative and positive ones. These are data quality, the baseline problem, our method of measuring BP onset, our method of measuring BPs forward from the preceding movement, and our reporting of behavioral parameters.

Data quality turned out to be a bigger problem than anticipated. The experiments reported here took place amidst experiments on the P3 component where stimulus-evoked potentials were being measured for intervals of 1.5 s, with generally good data quality (e.g., Verleger et al., 2015; Verleger and Śmigasiewicz, 2016). But here, with recorded intervals of 6 s and more, we had to struggle with the ever-present DC drifts in the data, even after applying our usual criteria for rejecting artifacts (and, not reported in Methods, after unsatisfactory results of attempts to isolate and eliminate the drifts by means of Independent Component Analysis), such that we were forced to apply Brain Analyzer’s linear DC detrending procedure on our averaged pre-movement data. This means that the difference was minimized between the first 100 ms (5 s or 7 s before key-press) and the last 100 ms of the epoch (1 s after key-press). Being linear, the procedure does not alter the shape of the pre-movement negativity in relation to the entire epoch, but it certainly either leaves or adds some slow-frequency noise to the data. The noise problem might have been aggravated by our stipulation of having 50 trials per condition in Experiment 1. This might have been too few. As a result, 5 of the originally 25 participants had to be excluded due to poor data quality, and in order to test the major question about differences between required minimum intervals, data were pooled across the count and no-count conditions. Based on this experience, we raised the required number of key-presses per condition to 100 in Experiment 2. It is comforting that, in spite of the presumably poorer signal/noise ratio in Experiment 1, significant differences in BP parameters were obtained in Experiment 1 but not in Experiment 2. Actually, the grand means of the no-count and count instructions (Figure 4) do not nourish much hope that the absence of a difference between these conditions is a problem of noise and lacking power. Not a hint of a difference between instructions is seen in the relatively smooth 2 s and 1 s waveforms. It is the noisier waveforms of the longer-interval conditions (lower panel of Figure 4)—5 s and particularly 3 s—that may suggest that the no-count instruction induces larger BPs. This does not appear to be reliable. One might suggest, though, that the final word has not been spoken yet, since any differences between these instructions might be more distinct when minimum intervals are longer (here 3 s and 5 s minimum). Therefore, the comparison between these instructions should be replicated with averages that include more trials than the maximum of 50 trials used in the present study.

A difficult problem in Experiment 1 was where to set the baseline in order to achieve a fair comparison between the experimental conditions with their differently shaped BP waveforms. It is obvious from Figure 2 that there were systematic fluctuations in the BP waveforms long before the rise of the present BP. In particular, around the average time-points of the preceding key-press (indicated by the arrowheads) there was slow phasic negativity followed by a positive downturn. This is most probably the preceding BP peak followed by the positive downturn after movement onset, jittered in time due to the variable time intervals between present and preceding movements. Finding a neutral baseline proved difficult under this condition. One has to keep in mind that these waveforms also differ between recording sites and that, therefore, choice of baselines will inevitably alter the topographical distributions of any voltages measured against baseline (Urbach and Kutas, 2006). This may be a reason why the scalp topographies of our BP peaks (Figure 2) appear to be more posteriorly focused than usual (e.g., Cui et al., 1999; Schultze-Kraft et al., 2016). One might also suspect that this was due to our reference placed at the nose rather than at the more commonly used mastoids. But when we re-referenced our data to TP7 and TP8, midline topography did not appreciably change. Choice of baseline likewise was a difficult problem in the post-movement potentials. We decided for a time when the positive post-movement potential gave way to the newly arising BP (0.7–0.9 ms after key-press). This made sense because then the BP slope was similar in all conditions. But obviously this was a critical decision.

We are not aware of any other study where BP onsets were illustrated and analyzed in temporal relation to the preceding movement in the way we did this in Experiment 1 (lower panel of Figure 2). Yet this might be an interesting parameter for example in studies on patients (for review see Verleger, 2004). It may be interesting to know whether, e.g., patients with Parkinson’s disease will have similar smooth transitions as healthy persons from executing one movement to preparing the next one.

Also worth discussing is our method of measuring BP onset. There are careful studies on how onsets of movement-related potentials should be measured, focusing on the “lateralized readiness potential” (LRP), i.e., the small difference in negativity between contralateral and ipsilateral motor cortex before hand movements (Miller et al., 1998; Mordkoff and Gianaros, 2000). Based on simulated data, Miller et al. (1998) (e.g., also Kiesel et al., 2008) recommended threshold values for measuring peak amplitudes, expressed in percentages of peak values. Thresholds of 50% were recommended for determining the onset of response-locked potentials, as a safeguard against noise. However, we may note that these thresholds were applied forward in time, denoting the first exceeding of threshold, whereupon the potential might fluctuate below threshold again. This is why the high value of 50% was needed. In contrast, we searched for the final exceeding of the threshold or, in other words, we searched backwards in time from movement onset for the first decrease of BP below threshold. This is why a threshold as low as 20% made sense. We acknowledge that this intuitively plausible method should be evaluated by simulated data similarly to what had been done by Miller et al. (1998) and Mordkoff and Gianaros (2000).

Finally, we would like to propose that behavioral parameters be routinely reported in BP studies, as we did here, i.e., means and variabilities of intervals between the required movements. This might be particularly important when comparing some movement-impaired patient groups to healthy participants because any abnormalities in BP might be normal consequences of abnormal behavior rather than neurophysiological abnormalities in the presence of normal behavior.

To conclude, BPs did vary in accordance with the temporal constraints on the intervals between movements, but we could not provide evidence for the notion that the process reflected by BPs is the timing mechanism. Of interest, our data did provide another piece of evidence that the process reflected by BP is not an automatic movement trigger (Deecke and Soekadar, 2016; Schultze-Kraft et al., 2016): if reflecting some brain process necessary and sufficient for movement, unrelated to timing, the BP would have started at the same time before movements, would start at staggered times after movements depending on the required minimum time intervals, and would reach equal amplitudes before movement onsets irrespective of the required interval between movements.

## Author Contributions

RV devised the experiments and wrote the manuscript, MH conducted Experiment 1 and contributed parts of the manuscript, AB conducted Experiment 2, KŚ supervised the experiments and critically discussed the manuscript. All authors have approved the final version.

## Conflict of Interest Statement

The authors declare that the research was conducted in the absence of any commercial or financial relationships that could be construed as a potential conflict of interest.

## Footnotes

^1^www.easycap.de

^2^www.brainproducts.com
